# Ethyl 1-*tert*-butyl-2-(4-hydr­oxy-3-methoxy­phen­yl)-1*H*-benzimidazole-5-carboxyl­ate

**DOI:** 10.1107/S1600536810011918

**Published:** 2010-04-10

**Authors:** Natarajan Arumugam, Aisyah Saad Abdul Rahim, Hasnah Osman, Mohd Mustaqim Rosli, Hoong-Kun Fun

**Affiliations:** a School of Pharmaceutical Sciences, Universiti Sains Malaysia, 11800 USM, Penang, Malaysia; b School of Chemical Sciences, Universiti Sains Malaysia, 11800 USM, Penang, Malaysia; c X-ray Crystallography Unit, School of Physics, Universiti Sains Malaysia, 11800 USM, Penang, Malaysia

## Abstract

In the title compound, C_21_H_24_N_2_O_4_, the benzimidazole ring system is almost planar, with a maximum deviation of 0.047 (1) Å and makes a dihedral angle of 88.44 (5)° with the attached benzene ring. In the crystal, mol­ecules form infinite chains along the *b* axis by way of inter­molecular O—H⋯N and C—H⋯O inter­actions. Weak C—H⋯π also contribute to the stabilization of the crystal structure.

## Related literature

For the biological properties of benzimidazole-based heterocyclic compounds, see: Townsend *et al.* (1970 ▶); Blythin *et al.* (1986 ▶); Lemura *et al.* (1986 ▶); Zhang *et al.* (2008 ▶); Bonfanti *et al.* (2008 ▶); Ozden *et al.* (2008 ▶). For related structures, see Arumugam, Abd Hamid *et al.* (2010 ▶); Arumugam, Abdul Rahim, Abd Hamid *et al.* (2010 ▶); Arumugam, Abdul Rahim, Osman *et al.* (2010 ▶). For the stability of the temperature controller used in the data collection, see: Cosier & Glazer (1986 ▶).
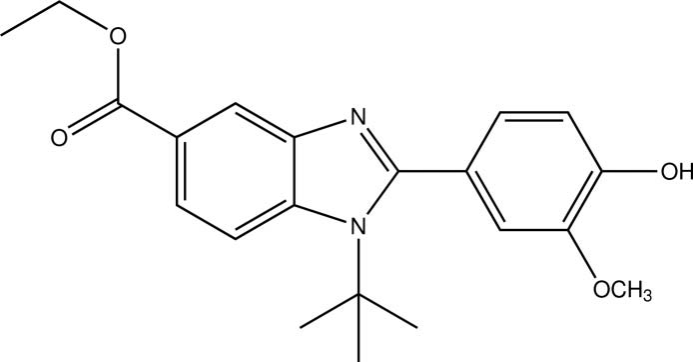


## Experimental

### 

#### Crystal data

C_21_H_24_N_2_O_4_
*M* *_r_* = 368.42
Monoclinic, 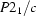

*a* = 9.2610 (6) Å
*b* = 13.6096 (9) Å
*c* = 16.3200 (9) Å
β = 113.560 (3)°
*V* = 1885.5 (2) Å^3^
*Z* = 4
Mo *K*α radiation
μ = 0.09 mm^−1^
*T* = 100 K
0.38 × 0.23 × 0.15 mm

#### Data collection

Bruker SMART APEXII CCD area-detector diffractometer
Absorption correction: multi-scan (*SADABS*; Bruker, 2005 ▶) *T*_min_ = 0.967, *T*_max_ = 0.986
21041 measured reflections
5484 independent reflections
4104 reflections with *I* > 2σ(*I*)
*R*_int_ = 0.039

#### Refinement

*R*[*F* ^2^ > 2σ(*F* ^2^)] = 0.047
*wR*(*F* ^2^) = 0.134
*S* = 1.07
5484 reflections
253 parameters
H atoms treated by a mixture of independent and constrained refinement
Δρ_max_ = 0.36 e Å^−3^
Δρ_min_ = −0.38 e Å^−3^

### 

Data collection: *APEX2* (Bruker, 2005 ▶); cell refinement: *SAINT* (Bruker, 2005 ▶); data reduction: *SAINT*; program(s) used to solve structure: *SHELXTL* (Sheldrick, 2008 ▶); program(s) used to refine structure: *SHELXTL*; molecular graphics: *SHELXTL*; software used to prepare material for publication: *SHELXTL* and *PLATON* (Spek, 2009 ▶).

## Supplementary Material

Crystal structure: contains datablocks global, I. DOI: 10.1107/S1600536810011918/wn2380sup1.cif

Structure factors: contains datablocks I. DOI: 10.1107/S1600536810011918/wn2380Isup2.hkl

Additional supplementary materials:  crystallographic information; 3D view; checkCIF report

## Figures and Tables

**Table 1:** Hydrogen-bond geometry (Å, °) *Cg*1 is the centroid of the C8–C13 benzene ring.

*D*—H⋯*A*	*D*—H	H⋯*A*	*D*⋯*A*	*D*—H⋯*A*
O1—H1*O*1⋯N1^i^	0.85 (2)	1.97 (2)	2.7475 (17)	151.7 (19)
C2—H2*A*⋯O2^ii^	0.93	2.58	3.3648 (17)	142
C13—H13*A*⋯O3^iii^	0.93	2.56	3.4223 (17)	154
C18—H18*B*⋯O4^iii^	0.96	2.60	3.514 (2)	160
C18—H18*C*⋯O1^iv^	0.96	2.54	3.4753 (17)	163
C18—H18*A*⋯*Cg*1^v^	0.96	2.89	3.5798 (16)	129
C20—H20*B*⋯*Cg*1	0.96	2.81	3.4764 (17)	127

Symmetry codes: (i) 
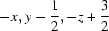
; (ii) 
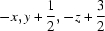
; (iii) 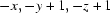; (iv) 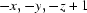; (v) 
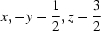
.

**Fig. 1.**
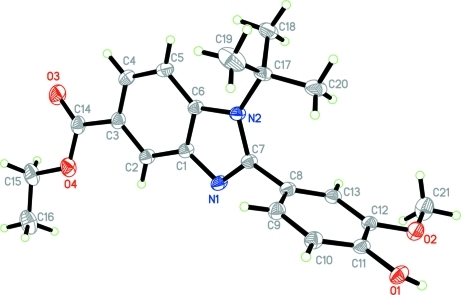
The molecular structure of the title compound, showing 50% probability displacement ellipsoids and the atom-numbering scheme. Hydrogen atoms are shown as spheres of arbitrary radius.

**Fig. 2.**
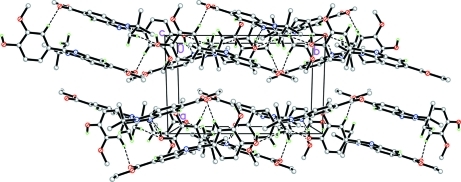
The crystal structure of the title compound, showing infinite chains along the b-axis. Hydrogen bonds are shown as dashed lines. H atoms not involved in these interactions have been omitted for clarity.

## supplementary crystallographic information

### Comment

Benzimidazole-based heterocycles are known to exhibit antihistamine (Lemura *et al.*,  1986),  immunosuppressive (Zhang *et al.*, 2008), anti-inflammatory (Blythin *et al.*,  1986), antiviral (Bonfanti *et al.*,  2008) and antibacterial (Ozden *et al.*,  2008) activities. In particular, substituted benzimidazole derivatives act as potential anticancer agents (Townsend *et al.*, 1970). In view of their importance, the crystal structure determination of the title compound was carried out and the results are presented here.

All molecular geometric parameters in the title compound are within normal ranges and are comparable with those in related crystal structures (Arumugam, Abd Hamid *et al.* (2010); Arumugam, Abdul Rahim, Abd Hamid *et al.* (2010); Arumugam, Abdul Rahim, Osman *et al.* (2010)). The benzimidazole ring system (N1/N2/C1-C7) is planar with a maximum deviation of 0.047 (1) Å for atom C1. The dihedral angle between the benzimidazole ring system (N1/N2/C1-C7) and the attached benzene ring (C8-C13) is 88.44 (5)°.

In the crystal structure, molecules are connected by intermolecluar O1—H1O1···N1^i^, C2—H2A···O2^ii^, C13—H13A···O3^iii^, C18—H18B···O4^iii^ and C18—H18C···O1^iv^ interactions (Table 1). These interactions link the molecules to form infinite one-dimensional chains along the b-axis (Fig. 2). The crystal structure is further stabilized by C—H···π interactions (Table 1), involving the C8-C13 (centroid Cg1) rings.

### Experimental

The title compound was synthesised according to the previous procedure described by us (Arumugam, Abd Hamid *et al.* (2010); Arumugam, Abdul Rahim, Abd Hamid *et al.* (2010); Arumugam, Abdul Rahim, Osman *et al.* (2010)). The product was recrystallised from EtOAc to afford the title compound as colourless crystals.

### Refinement

The H atom attached to O1 was located in a difference map and refined isotropically. The remaining H atoms were positioned geometrically [CH = 0.93, 0.96 or 0.97 Å] and were refined using a riding model, with *U*_iso_(H) = x*U*_eq_(C), where x = 1.5 for methyl H and 1.2 for all other H atoms. A rotating group model was used for the methyl groups.

### Crystal data

**Table:**

C_21_H_24_N_2_O_4_	*F*(000) = 784
*M**_r_* = 368.42	*D*_x_ = 1.298 Mg m^−^^3^
Monoclinic, *P*2_1_/*c*	Mo *K*α radiation, λ = 0.71073 Å
Hall symbol: -P 2ybc	Cell parameters from 4617 reflections
*a* = 9.2610 (6) Å	θ = 2.4–30.0°
*b* = 13.6096 (9) Å	µ = 0.09 mm^−^^1^
*c* = 16.3200 (9) Å	*T* = 100 K
β = 113.560 (3)°	Block, colourless
*V* = 1885.5 (2) Å^3^	0.38 × 0.23 × 0.15 mm
*Z* = 4	

### Data collection

**Table:**

Bruker SMART APEXII CCD area-detector diffractometer	5484 independent reflections
Radiation source: fine-focus sealed tube	4104 reflections with *I* > 2σ(*I*)
graphite	*R*_int_ = 0.039
φ and ω scans	θ_max_ = 30.0°, θ_min_ = 2.0°
Absorption correction: multi-scan (*SADABS*; Bruker, 2005)	*h* = −13→13
*T*_min_ = 0.967, *T*_max_ = 0.986	*k* = −16→19
21041 measured reflections	*l* = −22→22

### Refinement

**Table:**

Refinement on *F*^2^	Primary atom site location: structure-invariant direct methods
Least-squares matrix: full	Secondary atom site location: difference Fourier map
*R*[*F*^2^ > 2σ(*F*^2^)] = 0.047	Hydrogen site location: inferred from neighbouring sites
*wR*(*F*^2^) = 0.134	H atoms treated by a mixture of independent and constrained refinement
*S* = 1.07	*w* = 1/[σ^2^(*F*_o_^2^) + (0.0645*P*)^2^ + 0.3983*P*] where *P* = (*F*_o_^2^ + 2*F*_c_^2^)/3
5484 reflections	(Δ/σ)_max_ < 0.001
253 parameters	Δρ_max_ = 0.36 e Å^−^^3^
0 restraints	Δρ_min_ = −0.38 e Å^−^^3^

### Special details

**Table:**

Experimental. The crystal was placed in the cold stream of an Oxford Cyrosystems Cobra open-flow nitrogen cryostat (Cosier & Glazer, 1986) operating at 100.0 (1) K.
Geometry. All esds (except the esd in the dihedral angle between two l.s. planes) are estimated using the full covariance matrix. The cell esds are taken into account individually in the estimation of esds in distances, angles and torsion angles; correlations between esds in cell parameters are only used when they are defined by crystal symmetry. An approximate (isotropic) treatment of cell esds is used for estimating esds involving l.s. planes.
Refinement. Refinement of F^2^ against ALL reflections. The weighted R-factor wR and goodness of fit S are based on F^2^, conventional R-factors R are based on F, with F set to zero for negative F^2^. The threshold expression of F^2^ > 2sigma(F^2^) is used only for calculating R-factors(gt) etc. and is not relevant to the choice of reflections for refinement. R-factors based on F^2^ are statistically about twice as large as those based on F, and R- factors based on ALL data will be even larger.

### Fractional atomic coordinates and isotropic or equivalent isotropic displacement parameters (Å^2^)

**Table:**

	*x*	*y*	*z*	*U*_iso_*/*U*_eq_	
O1	0.07059 (12)	−0.06665 (7)	0.77515 (6)	0.0237 (2)	
O2	−0.19758 (11)	0.02786 (7)	0.67345 (6)	0.0254 (2)	
O3	0.35717 (13)	0.74808 (8)	0.53807 (7)	0.0364 (3)	
O4	0.36828 (12)	0.71957 (7)	0.67609 (7)	0.0287 (2)	
N1	0.14388 (13)	0.37334 (8)	0.65777 (7)	0.0217 (2)	
N2	0.14885 (12)	0.29733 (8)	0.53582 (7)	0.0188 (2)	
C1	0.18878 (14)	0.44031 (9)	0.60878 (8)	0.0197 (2)	
C2	0.23604 (15)	0.53759 (9)	0.63023 (9)	0.0216 (3)	
H2A	0.2337	0.5669	0.6812	0.026*	
C3	0.28664 (15)	0.58934 (10)	0.57327 (9)	0.0226 (3)	
C4	0.28918 (17)	0.54393 (11)	0.49670 (9)	0.0280 (3)	
H4A	0.3209	0.5803	0.4586	0.034*	
C5	0.24598 (17)	0.44675 (11)	0.47600 (9)	0.0266 (3)	
H5A	0.2513	0.4171	0.4259	0.032*	
C6	0.19403 (14)	0.39470 (9)	0.53306 (8)	0.0189 (2)	
C7	0.12336 (15)	0.28957 (9)	0.61386 (8)	0.0191 (2)	
C8	0.10037 (15)	0.19642 (9)	0.65461 (8)	0.0200 (2)	
C9	0.23493 (16)	0.14552 (10)	0.70872 (9)	0.0230 (3)	
H9A	0.3338	0.1700	0.7176	0.028*	
C10	0.22263 (15)	0.05846 (10)	0.74952 (8)	0.0225 (3)	
H10A	0.3135	0.0253	0.7859	0.027*	
C11	0.07666 (15)	0.02047 (9)	0.73664 (8)	0.0200 (2)	
C12	−0.05955 (14)	0.07243 (9)	0.68283 (8)	0.0194 (2)	
C13	−0.04751 (15)	0.16086 (9)	0.64290 (8)	0.0208 (2)	
H13A	−0.1378	0.1959	0.6086	0.025*	
C14	0.34014 (15)	0.69310 (10)	0.59183 (10)	0.0259 (3)	
C15	0.42106 (18)	0.82010 (11)	0.70149 (11)	0.0345 (3)	
H15A	0.3440	0.8664	0.6631	0.041*	
H15B	0.5206	0.8316	0.6966	0.041*	
C16	0.43956 (19)	0.83178 (12)	0.79658 (12)	0.0391 (4)	
H16A	0.4813	0.8958	0.8179	0.059*	
H16B	0.5104	0.7825	0.8330	0.059*	
H16C	0.3388	0.8245	0.7998	0.059*	
C17	0.13687 (15)	0.22114 (10)	0.46624 (8)	0.0216 (3)	
C18	0.03674 (17)	0.26257 (11)	0.37384 (9)	0.0288 (3)	
H18A	0.0878	0.3193	0.3627	0.043*	
H18B	−0.0649	0.2809	0.3715	0.043*	
H18C	0.0244	0.2135	0.3292	0.043*	
C19	0.30259 (19)	0.19690 (15)	0.47440 (12)	0.0442 (4)	
H19A	0.3547	0.2562	0.4694	0.066*	
H19B	0.2968	0.1524	0.4276	0.066*	
H19C	0.3608	0.1669	0.5314	0.066*	
C20	0.0556 (2)	0.12740 (11)	0.47674 (10)	0.0371 (4)	
H20A	−0.0449	0.1436	0.4774	0.056*	
H20B	0.1198	0.0956	0.5318	0.056*	
H20C	0.0406	0.0840	0.4276	0.056*	
C21	−0.34028 (17)	0.07449 (12)	0.61686 (11)	0.0351 (3)	
H21A	−0.4283	0.0351	0.6138	0.053*	
H21B	−0.3418	0.0818	0.5580	0.053*	
H21C	−0.3470	0.1381	0.6406	0.053*	
H1O1	−0.017 (2)	−0.0742 (15)	0.7803 (13)	0.043 (5)*	

### Atomic displacement parameters (Å^2^)

**Table:**

	*U*^11^	*U*^22^	*U*^33^	*U*^12^	*U*^13^	*U*^23^
O1	0.0303 (5)	0.0185 (5)	0.0257 (5)	0.0017 (4)	0.0147 (4)	0.0052 (4)
O2	0.0234 (4)	0.0252 (5)	0.0272 (5)	−0.0011 (4)	0.0097 (4)	0.0039 (4)
O3	0.0399 (6)	0.0290 (6)	0.0354 (6)	−0.0100 (4)	0.0098 (5)	0.0102 (4)
O4	0.0335 (5)	0.0167 (5)	0.0321 (5)	−0.0054 (4)	0.0090 (4)	−0.0001 (4)
N1	0.0333 (6)	0.0147 (5)	0.0223 (5)	−0.0016 (4)	0.0165 (4)	−0.0007 (4)
N2	0.0254 (5)	0.0159 (5)	0.0167 (5)	0.0003 (4)	0.0102 (4)	−0.0001 (4)
C1	0.0244 (6)	0.0160 (6)	0.0209 (6)	0.0017 (4)	0.0113 (5)	0.0014 (4)
C2	0.0278 (6)	0.0160 (6)	0.0233 (6)	0.0006 (5)	0.0127 (5)	0.0007 (5)
C3	0.0225 (6)	0.0192 (6)	0.0248 (6)	−0.0014 (5)	0.0082 (5)	0.0035 (5)
C4	0.0327 (7)	0.0291 (7)	0.0236 (6)	−0.0071 (6)	0.0129 (5)	0.0047 (5)
C5	0.0345 (7)	0.0286 (7)	0.0198 (6)	−0.0048 (6)	0.0141 (5)	0.0004 (5)
C6	0.0211 (5)	0.0180 (6)	0.0181 (5)	0.0006 (4)	0.0084 (4)	0.0019 (4)
C7	0.0257 (6)	0.0158 (6)	0.0180 (5)	0.0010 (5)	0.0109 (5)	−0.0001 (4)
C8	0.0308 (6)	0.0139 (5)	0.0175 (5)	−0.0014 (5)	0.0121 (5)	−0.0018 (4)
C9	0.0269 (6)	0.0194 (6)	0.0239 (6)	−0.0012 (5)	0.0115 (5)	0.0002 (5)
C10	0.0254 (6)	0.0199 (6)	0.0221 (6)	0.0022 (5)	0.0096 (5)	0.0019 (5)
C11	0.0292 (6)	0.0156 (6)	0.0170 (5)	0.0004 (5)	0.0112 (5)	−0.0009 (4)
C12	0.0246 (6)	0.0179 (6)	0.0171 (5)	−0.0007 (5)	0.0096 (4)	−0.0017 (4)
C13	0.0278 (6)	0.0174 (6)	0.0174 (5)	0.0010 (5)	0.0093 (5)	−0.0001 (4)
C14	0.0224 (6)	0.0209 (6)	0.0305 (7)	−0.0021 (5)	0.0066 (5)	0.0057 (5)
C15	0.0337 (7)	0.0170 (6)	0.0434 (9)	−0.0045 (5)	0.0055 (6)	0.0015 (6)
C16	0.0315 (7)	0.0257 (8)	0.0512 (10)	0.0000 (6)	0.0072 (7)	−0.0070 (7)
C17	0.0282 (6)	0.0203 (6)	0.0174 (5)	0.0023 (5)	0.0102 (5)	−0.0032 (5)
C18	0.0363 (7)	0.0271 (7)	0.0188 (6)	−0.0016 (6)	0.0066 (5)	−0.0020 (5)
C19	0.0322 (8)	0.0571 (11)	0.0409 (9)	0.0112 (7)	0.0119 (7)	−0.0200 (8)
C20	0.0704 (11)	0.0197 (7)	0.0252 (7)	−0.0065 (7)	0.0235 (7)	−0.0064 (5)
C21	0.0242 (6)	0.0335 (8)	0.0437 (9)	0.0012 (6)	0.0095 (6)	0.0084 (7)

### Geometric parameters (Å, °)

**Table:**

O1—C11	1.3536 (15)	C10—C11	1.3827 (18)
O1—H1O1	0.857 (19)	C10—H10A	0.9300
O2—C12	1.3672 (15)	C11—C12	1.4060 (17)
O2—C21	1.4235 (17)	C12—C13	1.3941 (17)
O3—C14	1.2104 (17)	C13—H13A	0.9300
O4—C14	1.3424 (18)	C15—C16	1.500 (2)
O4—C15	1.4563 (17)	C15—H15A	0.9700
N1—C7	1.3193 (16)	C15—H15B	0.9700
N1—C1	1.3815 (16)	C16—H16A	0.9600
N2—C7	1.3890 (16)	C16—H16B	0.9600
N2—C6	1.3958 (16)	C16—H16C	0.9600
N2—C17	1.5086 (16)	C17—C19	1.523 (2)
C1—C2	1.3945 (17)	C17—C20	1.525 (2)
C1—C6	1.4010 (17)	C17—C18	1.5278 (18)
C2—C3	1.3885 (18)	C18—H18A	0.9600
C2—H2A	0.9300	C18—H18B	0.9600
C3—C4	1.4027 (19)	C18—H18C	0.9600
C3—C14	1.4871 (19)	C19—H19A	0.9600
C4—C5	1.384 (2)	C19—H19B	0.9600
C4—H4A	0.9300	C19—H19C	0.9600
C5—C6	1.3995 (18)	C20—H20A	0.9600
C5—H5A	0.9300	C20—H20B	0.9600
C7—C8	1.4858 (17)	C20—H20C	0.9600
C8—C9	1.3903 (18)	C21—H21A	0.9600
C8—C13	1.3918 (18)	C21—H21B	0.9600
C9—C10	1.3860 (18)	C21—H21C	0.9600
C9—H9A	0.9300		
			
C11—O1—H1O1	111.5 (13)	O3—C14—O4	123.26 (13)
C12—O2—C21	117.31 (11)	O3—C14—C3	124.66 (14)
C14—O4—C15	116.70 (11)	O4—C14—C3	112.08 (11)
C7—N1—C1	105.49 (10)	O4—C15—C16	106.30 (12)
C7—N2—C6	105.36 (10)	O4—C15—H15A	110.5
C7—N2—C17	130.51 (11)	C16—C15—H15A	110.5
C6—N2—C17	124.12 (10)	O4—C15—H15B	110.5
N1—C1—C2	128.17 (12)	C16—C15—H15B	110.5
N1—C1—C6	110.05 (11)	H15A—C15—H15B	108.7
C2—C1—C6	121.60 (12)	C15—C16—H16A	109.5
C3—C2—C1	118.01 (12)	C15—C16—H16B	109.5
C3—C2—H2A	121.0	H16A—C16—H16B	109.5
C1—C2—H2A	121.0	C15—C16—H16C	109.5
C2—C3—C4	120.21 (12)	H16A—C16—H16C	109.5
C2—C3—C14	121.30 (12)	H16B—C16—H16C	109.5
C4—C3—C14	118.49 (12)	N2—C17—C19	108.35 (11)
C5—C4—C3	122.17 (13)	N2—C17—C20	112.42 (10)
C5—C4—H4A	118.9	C19—C17—C20	109.53 (13)
C3—C4—H4A	118.9	N2—C17—C18	108.80 (11)
C4—C5—C6	117.65 (13)	C19—C17—C18	111.05 (12)
C4—C5—H5A	121.2	C20—C17—C18	106.70 (11)
C6—C5—H5A	121.2	C17—C18—H18A	109.5
N2—C6—C5	133.58 (12)	C17—C18—H18B	109.5
N2—C6—C1	105.99 (10)	H18A—C18—H18B	109.5
C5—C6—C1	120.33 (12)	C17—C18—H18C	109.5
N1—C7—N2	113.08 (11)	H18A—C18—H18C	109.5
N1—C7—C8	120.63 (11)	H18B—C18—H18C	109.5
N2—C7—C8	125.63 (11)	C17—C19—H19A	109.5
C9—C8—C13	119.80 (12)	C17—C19—H19B	109.5
C9—C8—C7	117.19 (11)	H19A—C19—H19B	109.5
C13—C8—C7	122.98 (11)	C17—C19—H19C	109.5
C10—C9—C8	120.41 (12)	H19A—C19—H19C	109.5
C10—C9—H9A	119.8	H19B—C19—H19C	109.5
C8—C9—H9A	119.8	C17—C20—H20A	109.5
C11—C10—C9	120.65 (12)	C17—C20—H20B	109.5
C11—C10—H10A	119.7	H20A—C20—H20B	109.5
C9—C10—H10A	119.7	C17—C20—H20C	109.5
O1—C11—C10	118.45 (11)	H20A—C20—H20C	109.5
O1—C11—C12	122.49 (11)	H20B—C20—H20C	109.5
C10—C11—C12	119.04 (12)	O2—C21—H21A	109.5
O2—C12—C13	125.21 (11)	O2—C21—H21B	109.5
O2—C12—C11	114.33 (11)	H21A—C21—H21B	109.5
C13—C12—C11	120.45 (12)	O2—C21—H21C	109.5
C8—C13—C12	119.62 (12)	H21A—C21—H21C	109.5
C8—C13—H13A	120.2	H21B—C21—H21C	109.5
C12—C13—H13A	120.2		
			
C7—N1—C1—C2	−174.69 (13)	C13—C8—C9—C10	1.40 (19)
C7—N1—C1—C6	0.37 (14)	C7—C8—C9—C10	179.51 (11)
N1—C1—C2—C3	175.59 (12)	C8—C9—C10—C11	0.46 (19)
C6—C1—C2—C3	1.04 (19)	C9—C10—C11—O1	177.51 (11)
C1—C2—C3—C4	0.06 (19)	C9—C10—C11—C12	−1.22 (19)
C1—C2—C3—C14	−179.51 (11)	C21—O2—C12—C13	1.43 (19)
C2—C3—C4—C5	−1.6 (2)	C21—O2—C12—C11	−177.52 (12)
C14—C3—C4—C5	177.98 (13)	O1—C11—C12—O2	0.46 (17)
C3—C4—C5—C6	2.0 (2)	C10—C11—C12—O2	179.14 (11)
C7—N2—C6—C5	174.74 (14)	O1—C11—C12—C13	−178.54 (11)
C17—N2—C6—C5	−4.1 (2)	C10—C11—C12—C13	0.14 (18)
C7—N2—C6—C1	−1.55 (13)	C9—C8—C13—C12	−2.46 (18)
C17—N2—C6—C1	179.58 (11)	C7—C8—C13—C12	179.55 (11)
C4—C5—C6—N2	−176.68 (13)	O2—C12—C13—C8	−177.19 (11)
C4—C5—C6—C1	−0.82 (19)	C11—C12—C13—C8	1.70 (18)
N1—C1—C6—N2	0.77 (14)	C15—O4—C14—O3	0.3 (2)
C2—C1—C6—N2	176.21 (11)	C15—O4—C14—C3	−179.85 (11)
N1—C1—C6—C5	−176.11 (12)	C2—C3—C14—O3	−165.35 (13)
C2—C1—C6—C5	−0.67 (19)	C4—C3—C14—O3	15.1 (2)
C1—N1—C7—N2	−1.44 (14)	C2—C3—C14—O4	14.78 (18)
C1—N1—C7—C8	169.72 (11)	C4—C3—C14—O4	−164.80 (12)
C6—N2—C7—N1	1.92 (14)	C14—O4—C15—C16	178.47 (12)
C17—N2—C7—N1	−179.30 (11)	C7—N2—C17—C19	−108.89 (16)
C6—N2—C7—C8	−168.71 (12)	C6—N2—C17—C19	69.69 (16)
C17—N2—C7—C8	10.1 (2)	C7—N2—C17—C20	12.30 (18)
N1—C7—C8—C9	−86.62 (15)	C6—N2—C17—C20	−169.12 (12)
N2—C7—C8—C9	83.36 (16)	C7—N2—C17—C18	130.27 (13)
N1—C7—C8—C13	91.42 (16)	C6—N2—C17—C18	−51.15 (16)
N2—C7—C8—C13	−98.60 (16)		

### Hydrogen-bond geometry (Å, °)

**Table:**

Cg1 is the centroid of the C8–C13 benzene ring.

**Table:**

*D*—H···*A*	*D*—H	H···*A*	*D*···*A*	*D*—H···*A*
O1—H1O1···N1^i^	0.85 (2)	1.97 (2)	2.7475 (17)	151.7 (19)
C2—H2A···O2^ii^	0.93	2.58	3.3648 (17)	142
C13—H13A···O3^iii^	0.93	2.56	3.4223 (17)	154
C18—H18B···O4^iii^	0.96	2.60	3.514 (2)	160
C18—H18C···O1^iv^	0.96	2.54	3.4753 (17)	163
C18—H18A···Cg1^v^	0.96	2.89	3.5798 (16)	129
C20—H20B···Cg1	0.96	2.81	3.4764 (17)	127

Symmetry codes: (i) −*x*, *y*−1/2, −*z*+3/2; (ii) −*x*, *y*+1/2, −*z*+3/2; (iii) −*x*, −*y*+1, −*z*+1; (iv) −*x*, −*y*, −*z*+1; (v) *x*, −*y*−1/2, *z*−3/2.
